# Deciphering Crossed Cerebellar Diaschisis in Patients with Large-Vessel Occlusion Acute Ischemic Stroke using BOLD Cerebrovascular Reactivity

**DOI:** 10.1007/s12311-026-02014-1

**Published:** 2026-05-07

**Authors:** Jacopo Bellomo, Vittorio Stumpo, Christiaan Hendrik Bas van Niftrik, Susanne Wegener, Andreas Luft, Zsolt Kulcsar, Luca Regli, Jorn Fierstra, Martina Sebök

**Affiliations:** 1 Department of Neurosurgery, University Hospital of Zurich, Frauenklinikstrasse 10, Zurich, CH-8091 Switzerland; 2 Clinical Neuroscience Center, University of Zurich, Zurich, Switzerland; 3 Department of Neurology, University Hospital of Zurich, Zurich, Switzerland; 4 Department of Neuroradiology, University Hospital of Zurich, Zurich, Switzerland; 5 Center for Neurology and Rehabilitation, Cereneo, Vitznau, Lucerne, Switzerland

**Keywords:** Stroke, BOLD-CVR, Metabolism, Hemodynamic, Crossed cerebellar diaschisis, CCD

## Abstract

Crossed cerebellar diaschisis (CCD) is characterized by reduced perfusion and metabolism in the cerebellar hemisphere contralateral to a supratentorial lesion. In large-vessel occlusion acute ischemic stroke (LVO-AIS), CCD may result from hemodynamic impairment, structural injury, or both. From a blood-oxygenation-level-dependent cerebrovascular reactivity (BOLD-CVR) imaging database, we identified patients with anterior-circulation LVO-AIS who underwent BOLD-CVR MRI within 7 days of symptom onset. Patients were stratified into those with persistent occlusion (non-endovascular thrombectomy, non-EVT) and those imaged after successful reperfusion (EVT). CCD was defined by a cerebellar asymmetry index > 12%. Associations between CCD and imaging markers of structural injury (infarct lesion volume) and hemodynamic impairment (steal phenomenon volume) as well as associations with 90-day functional outcome were assessed using logistic regression models. Sensitivity analyses included multiple imputation and best-/worst-case scenarios for missing outcomes. Seventy-nine patients were included (23 EVT, 56 non-EVT). CCD was present in 35% of EVT and 41% of non-EVT patients. In non-EVT patients, CCD was independently associated with larger steal phenomenon volumes (adjusted OR 1.99; 95% CI 1.12–3.73), but not infarct size. In EVT patients, CCD was associated with larger infarct lesions (adjusted OR 5.75; 95% CI 1.41–68.92) but not steal phenomenon volume. CCD predicted poorer 90-day outcome only in non-EVT patients in complete-case analysis, but this association was not robust in sensitivity analyses. CCD in acute LVO-AIS reflects different mechanisms depending on occlusion status: hemodynamic impairment under persistent occlusion and structural injury after reperfusion. BOLD-CVR imaging provides insight into CCD, though larger studies are needed to clarify its prognostic value.

**Supplementary Information:**

The online version contains supplementary material available at https://doi.org/10.1007/s12311-026-02014-1.

## Introduction

Crossed cerebellar diaschisis (CCD) is a neurophysiological and imaging phenomenon characterized by reduced blood flow and metabolism in the cerebellar hemisphere contralateral to a supratentorial brain lesion.^1^–^3^ First described in ischemic stroke by Baron et al. in 1981 4, CCD has gained increasing attention with the advent of advanced neuroimaging techniques.^1^–^3^.

Early studies investigating CCD in the chronic phase of supratentorial infarction used positron emission tomography (PET), single-photon emission computed tomography (SPECT), computed tomography (CT), and magnetic resonance (MR) perfusion imaging, linking CCD to degeneration of the cortico-cerebellar system secondary to supratentorial ischemic damage.^5^ However, PET and SPECT are limited by high costs and the need for radioisotopes. Blood oxygenation level-dependent (BOLD) cerebrovascular reactivity (CVR) imaging has recently emerged as a non-invasive MRI-based alternative using carbon dioxide (CO_2_) as a vasodilatory stimulus, with sensitivity comparable to (^15^O)-H_2_O-PET.^1^ In CCD, cerebellar hypometabolism results in reduced conversion of oxyhemoglobin to deoxyhemoglobin, leading to diminished deoxyhemoglobin washout during vasodilation and thus a blunted BOLD response.^2^,^6^.

Clinically, CCD has been associated with worse neurological status at admission and poorer functional outcome at 3 months after stroke.^2^ In patients with large-vessel occlusion acute ischemic stroke (LVO-AIS), CCD has been linked to the location and severity of supratentorial hypoperfusion, as well as infarct extent and distribution.^7^–^12^ Longitudinal data further suggest that CCD may be partially reversible after reperfusion, indicating both functional and structural mechanisms.^2^ We therefore hypothesized two potentially coexisting processes: (1) a functional, hemodynamic CCD driven by persistent hypoperfusion and reversible deafferentation of the cortico-cerebellar pathway; and (2) a structural, lesion-induced CCD resulting from irreversible infarct-related disruption of cortico-pontine fibers.^2^.

To disentangle these mechanisms, we studied two physiologically distinct and non-overlapping patient populations with LVO-AIS: patients with successful reperfusion after endovascular thrombectomy (EVT), representing a post-reperfusion state in which CCD predominantly reflects structural injury; and patients ineligible for EVT with persistent vessel occlusion, representing a hemodynamic state in which CCD primarily reflects ongoing perfusion impairment. The aim of this study was to investigate CCD detected by BOLD-CVR imaging in relation to structural injury (infarct lesion size) and hemodynamic impairment (steal phenomenon, i.e. negative BOLD-CVR values), assessed independently within each group.

## Materials and Methods

### Study Population

From our single-center, prospective BOLD-CVR database (June 2015–March 2024), we identified patients with acute ischemic stroke due to newly diagnosed anterior circulation large-vessel occlusion confirmed by CTA or MRA, who underwent BOLD-CVR imaging within 7 days of symptom onset. Patients were assessed by the institutional stroke team, and eligibility for EVT followed current guidelines.^13^ Detailed inclusion/exclusion criteria and acute management are provided in the **Supplemental Methods**.

Patients were stratified a priori into two independent groups: (1) EVT-ineligible patients with persistent LVO (non-EVT group), and (2) patients with successful EVT, defined as mTICI ≥2b 14 (EVT group). EVT-ineligible patients with large infarcts on baseline DWI were excluded. Patients with unsuccessful EVT (mTICI <2b) were excluded due to their intermediate hemodynamic state. In EVT-ineligible patients who later underwent extracranial-to-intracranial (EC-IC) bypass, BOLD-CVR imaging was performed prior to surgery. Clinical follow-up was 3 months.

### Ethics

The study was approved by the Cantonal Ethics Committee of Zurich (KEK-ZH-NR. 2012 − 0427, 2019–00750, 2020–02314). Written informed consent was obtained from all participants, and the study adhered to the Declaration of Helsinki.

### Image Acquisition

The imaging studies were performed using a 3-Tesla Skyra MRI scanner (Siemens Healthineers, Forchheim, Germany) with a 32-channel head matrix coil. BOLD-CVR acquisition details are provided in the **Supplemental Methods**.

### Image Processing

BOLD-CVR maps (%ΔBOLD/mmHg PetCO_2_) were generated using a previously validated analysis pipeline.^2^ After standard preprocessing, voxelwise BOLD-CVR values were calculated and co-registered to individual T1-weighted images. Mean BOLD-CVR values were extracted from predefined hemispheric, tissue-specific, and vascular territory regions of interest based on published atlases.^15^.

Hemodynamic impairment (steal phenomenon) was defined as negative BOLD-CVR values outside infarcted tissue and corresponding volumes were quantified within affected ACA and MCA territories^16^,^17^, reflecting areas in which blood flow paradoxically decreases during vasodilatory stimulation, indicating exhausted cerebrovascular reserve. The cerebellar asymmetry index (CAI) was computed as previously described.^1^,^2^ Ischemic lesions were automatically segmented from diffusion-weighted imaging using a validated deep learning algorithm^18^, and infarct voxels were excluded from all BOLD-CVR analyses. Detailed preprocessing steps, ROI definitions, and lesion segmentation procedures are provided in the **Supplemental Methods**.

### Outcome Measures

The primary outcome was CCD presence in the BOLD-CVR imaging, defined as CAI > 12%, corresponding to two standard deviations above the mean in healthy controls.^1^ The secondary outcome was functional status at 90 days, assessed by the modified Rankin Scale (mRS).

### Statistical Analysis

The statistical analysis was performed in RStudio (Posit Software, PBC formerly RStudio, version 2022-06-23) and conducted separately for non-EVT and EVT groups. BOLD-CVR metrics, steal phenomenon volume, and CAI were described for both patient groups. Associations between CCD and imaging variables (steal phenomenon volume, infarct volume) were evaluated using multivariable Firth’s penalized logistic regression adjusted for admission NIHSS and time between symptom onset and BOLD-CVR imaging. Further, functional outcome (90-day mRS) was analyzed using proportional-odds ordinal logistic regression, including CCD status, infarct volume, steal phenomenon volume, age, and admission mRS. The EVT model additionally included mTICI grade, while the non-EVT model included EC–IC bypass during follow-up. Model robustness was assessed using 1,000-sample bootstrap resampling. Missing 90-day mRS values (*n* = 13) were addressed with multiple imputation and best-/worst-case sensitivity analyses. Statistical significance was set at *p* < 0.05.

## Results

**Fig. 1:**
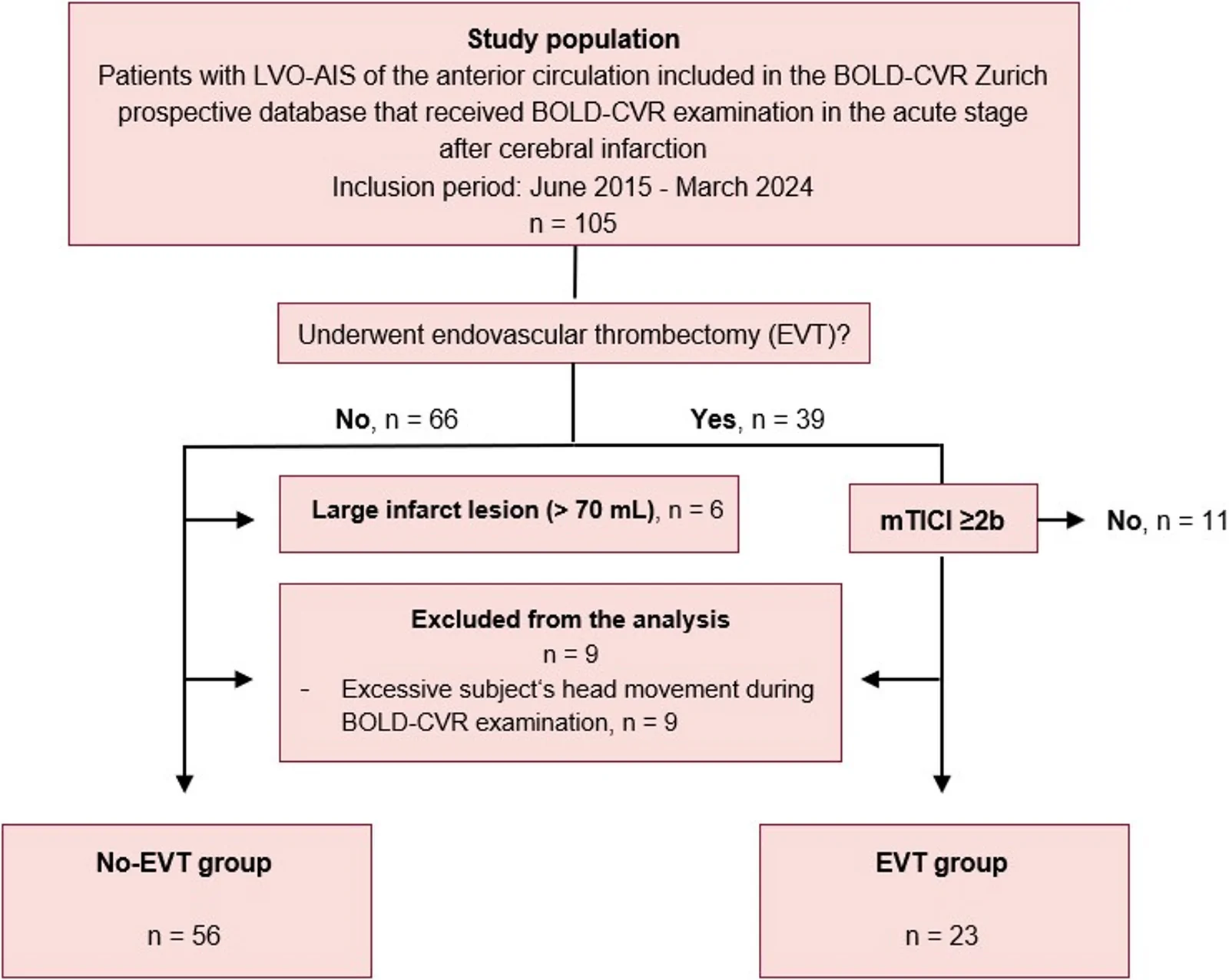
Study flow chart. BOLD-CVR, blood oxygenation-level dependent cerebrovascular reactivity; EVT, endovascular thrombectomy; LVO-AIS, large-vessel occlusion acute ischemic stroke; mTICI, modified Treatment In Cerebral Infarction

Between June 2015 and March 2024, 105 patients with anterior circulation LVO-AIS underwent acute-stage BOLD-CVR imaging. Among them, 39 patients received BOLD-CVR examination after EVT (EVT group), while 66 patients were not eligible for EVT (non-EVT group). Nine patients (5 in the EVT group and 4 in the non-EVT group) were excluded due to excessive head movement during MRI, 11 for unsuccessful EVT (mTICI <2b), and 6 EVT-ineligible patients for large baseline infarcts. The final cohort comprised 79 patients: 23 in the EVT group and 56 in the non-EVT group (Fig. 1; Table 1). During follow-up no deaths occurred; 13 patients (EVT *n* = 4; non-EVT *n* = 9) were lost to 90-day follow-up.

**Table 1:** Demographic and clinical baseline characteristics. CAD, coronary artery disease; CCD, crossed cerebellar diaschisis; EC-IC, extra-to-intracranial; EVT, endovascular thrombectomy; ICA, internal carotid artery; IQR, interquantile range MCA, middle cerebral artery; NA, not applicable; NIHSS, National Institutes of Health Stroke Scale; mRS, modified Rankin scale; PAD, peripheral artery disease; TIA, transient ischemic attack. ^**†**^*P* values are exploratory and should be interpreted accordingly. Between-group comparisons were conducted using the Pearson χ2 or Fisher exact test for categorical variables, and Student t test or Mann-Whitney U test for continuous variables respectively, as appropriate based on normality testing. *P value < 0.05

Baseline characteristics	EVT Group (*n* = 23)				Non-EVT Group (*n* = 56)				
	CCD (*n* = 8)	No CCD (*n* = 15)		*P* value^†^	CCD (*n* = 23)	No CCD (*n* = 33)			*P* value^†^
Age									
Median [IQR]	69.0 [67.5, 72.0]	66.0 [52.5, 82.5]		0.872	61.5 [57.7, 66.7]	69.0 [60.0, 76.0]			0.463
Sex									
M (%)	5 (62)	5 (33)		0.367	19 (83)	17 (52)			0.035*
F (%)	3 (38)	10 (67)			4 (17)	16 (48)			
Ischemic event									
Stroke (%)	8 (100)	15 (100)		NA	23 (100)	24 (73)			0.018*
TIA (%)	-	-			0 (0)	9 (27)			
Symptomatic side									
Left (%)	3 (37)	8 (53)		0.507	13 (57)	24 (73)			0.330
Right (%)	5 (63)	7 (47)			10 (43)	9 (27)			
Diseased vessel									
ICA (%)	1 (12)	2 (13)		0.515	19 (83)	24 (73)			0.576
MCA (M1/M2 segment) (%)	3 (38)	9 (60)			3 (13)	8 (24)			
Both (%)	4 (50)	4 (27)			1 (4)	1 (3)			
Acute treatment									
EVT (%)	8 (100)	15 (100)	NA		NA		NA	NA	
IVT (%)	3 (38)	9 (60)	1.000		1 (4)		2 (6)	1.000	
Clinical score at hospital admission									
NIHSS									
Median [IQR]	12.5 [9.7, 16.0]	10.0 [5.5, 15.5]		0.399	2.0 [1.0, 4.0]	0.0 [0.0, 2.0]			0.009*
Pre-stroke mRS									
Median [IQR]	4.0 [4.0, 5.0]	3.0 [2.0, 4.5]		0.143	2.0 [0.5, 2.5]	1.0 [0.0, 1.0]			0.027*
Comorbidities									
Atrial fibrillation (%)	2 (25)	2 (13)		0.900	2 (9)	4 (12)			1.000
Smoking history (%)	2 (25)	3 (20)		1.000	16 (70)	18 (55)			0.393
Hypertension (%)	4 (50)	10 (67)		0.740	17 (74)	28 (85)			0.502
Dyslipidemia (%)	3 (37)	6 (40)		1.000	8 (35)	25 (76)			0.005*
Obesity (%)	1 (13)	2 (13)		1.000	3 (13)	3 (9)			0.975
Diabetes (%)	0 (0)	1 (7)		1.000	4 (17)	13 (39)			0.143
CAD (%)	0 (0)	2 (13)		0.761	2 (9)	4 (12)			1.000
PAD (%)	0 (0)	1 (7)		1.000	2 (9)	5 (15)			0.758
Symptom-to-imaging time									
in hours, median [IQR]	42 [27, 59]	34 [26, 41]		0.671	98 [49, 129]	104 [69, 142]			0.748
Wake-up stroke (%)	4 (50)	3 (20)		0.311	NA	NA			NA
Follow-up time									
Additional intervention (%)									
- EC-IC bypass surgery	0 (0)	0 (0)		NA	10 (44)	4 (12)			0.019*
- None	8 (100)	15 (100)			13 (56)	29 (88)			
Lost to Follow-up (%)	1 (12)	3 (20)		0.925	5 (22)	4 (12)			0.875
Death (%)	0 (0)	0 (0)		NA	0 (0)	0 (0)			NA

**Fig. 2:**
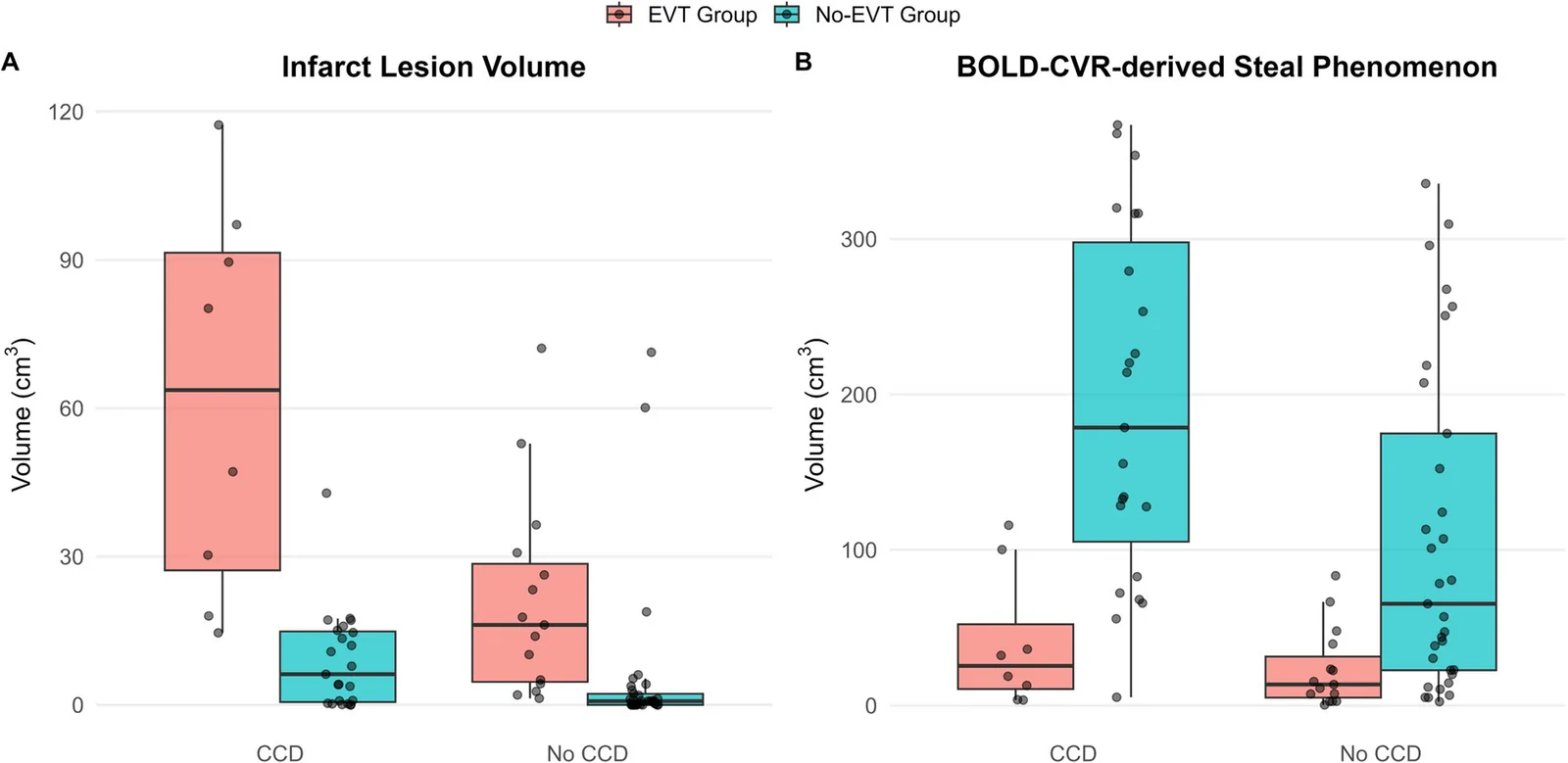
Distribution of morphological and hemodynamic imaging characteristics stratified by CCD status and EVT group. Boxplot comparison of morphological (infarct lesion volume) and hemodynamic (BOLD-CVR-derived steal phenomenon volume) imaging characteristics between patients with and without crossed cerebellar diaschisis (CCD). Each point represents an individual patient. Boxplots show median, interquartile range, and range after stratification by CCD status and EVT group. BOLD-CVR, blood oxygenation-level dependent cerebrovascular reactivity; CCD, crossed cerebellar diaschisis; DWI, diffusion-weighted imaging; EVT, endovascular thrombectomy

Baseline characteristics differed substantially between groups. Compared with EVT patients, the non-EVT group had lower admission stroke severity (NIHSS, median [IQR]: 1 [0, 3] vs. 10 [7, 16]), a higher prevalence of cardiovascular risk factors, and smaller infarct volumes on DWI (median [IQR]; 0.98 [0.13, 6.62] cm^3^ vs. 23.30 [12.03, 50.00] cm^3^; Table 1 and **Supplemental Table ****1**). CCD prevalence did not differ between groups (non-EVT: 23/56, 41% vs. EVT: 8/23, 35%; *p* = 0.44). The distribution of DWI-derived infarct volume and steal phenomenon volume by CCD status in both groups is illustrated in Fig. 2.

In the non-EVT group, CCD-positive patients showed lower mean BOLD-CVR values (0.00 ± 0.08 vs. 0.06 ± 0.06%ΔBOLD/mmHg; *p* = 0.003), larger steal phenomenon volumes (179 [105–298] vs. 65 [23–175] cm^3^; *p* = 0.002), and larger infarcts (6 [1–15] vs. 1 [0–2] cm^3^; *p* = 0.009) compared with CCD-negative patients (**Supplemental Table ****1**). In multivariable analysis, CCD was independently associated with steal phenomenon volume (adjusted OR 1.99, 95% CI 1.12–3.73; *p* = 0.018), but not infarct size (Table 2).

**Table 2:** Regression analysis of the association between infarct lesion size, steal phenomenon volume and CCD. Steal phenomenon volume encompasses the total volumes found within ACA and MCA vascular territories after subtracting the part included in the DWI infarct lesion. Steal phenomenon and infarct lesion size were scaled prior to regression model fitting. Regression analysis was performed separately in the EVT and non-EVT groups using Firth’s penalized logistic regression and adjusted for NIHSS at admission. BOLD-CVR indicates blood oxygenation-level dependent cerebrovascular reactivity; CCD, crossed cerebellar diaschisis; CI, confidence interval; DWI, diffusion-weighted imaging; EVT, endovascular thrombectomy; NIHSS, National Institutes of Health Stroke Scale; SOT, symptom onset; OR, odds ratio. *P value < 0.05

	Independent variable	Univariable OR (95% CI)	*P* value	Multivariable OR (95% CI)	*P* value
EVT Group	Steal phenomenon volume [cm^3^]	1.60 (0.73 to 3.95)	0.24	0.47 (0.07 to 1.78)	0.29
	DWI-derived infarct lesion volume [cm^3^]	3.59 (1.37 to 14.47)	0.007*	5.27 (1.30 to 58.80)	0.017*
	NIHSS at admission			1.01 (0.81 to 1.27)	0.90
	Time between SOT and BOLD-CVR MRI			1.19 (0.48 to 2.93)	0.68
Non-EVT Group	Steal phenomenon volume [cm^3^]	2.18 (1.25 to 4.06)	0.006*	1.96 (1.11 to 3.68)	0.019*
	DWI-derived infarct lesion volume [cm^3^]	1.22 (0.76 to 2.18)	0.40	1.02 (0.55 to 1.79)	0.92
	NIHSS at admission			1.16 (0.93 to 1.50)	0.17
	Time between SOT and BOLD-CVR MRI			0.86 (0.47 to 1.54)	0.62

**Fig. 3:**
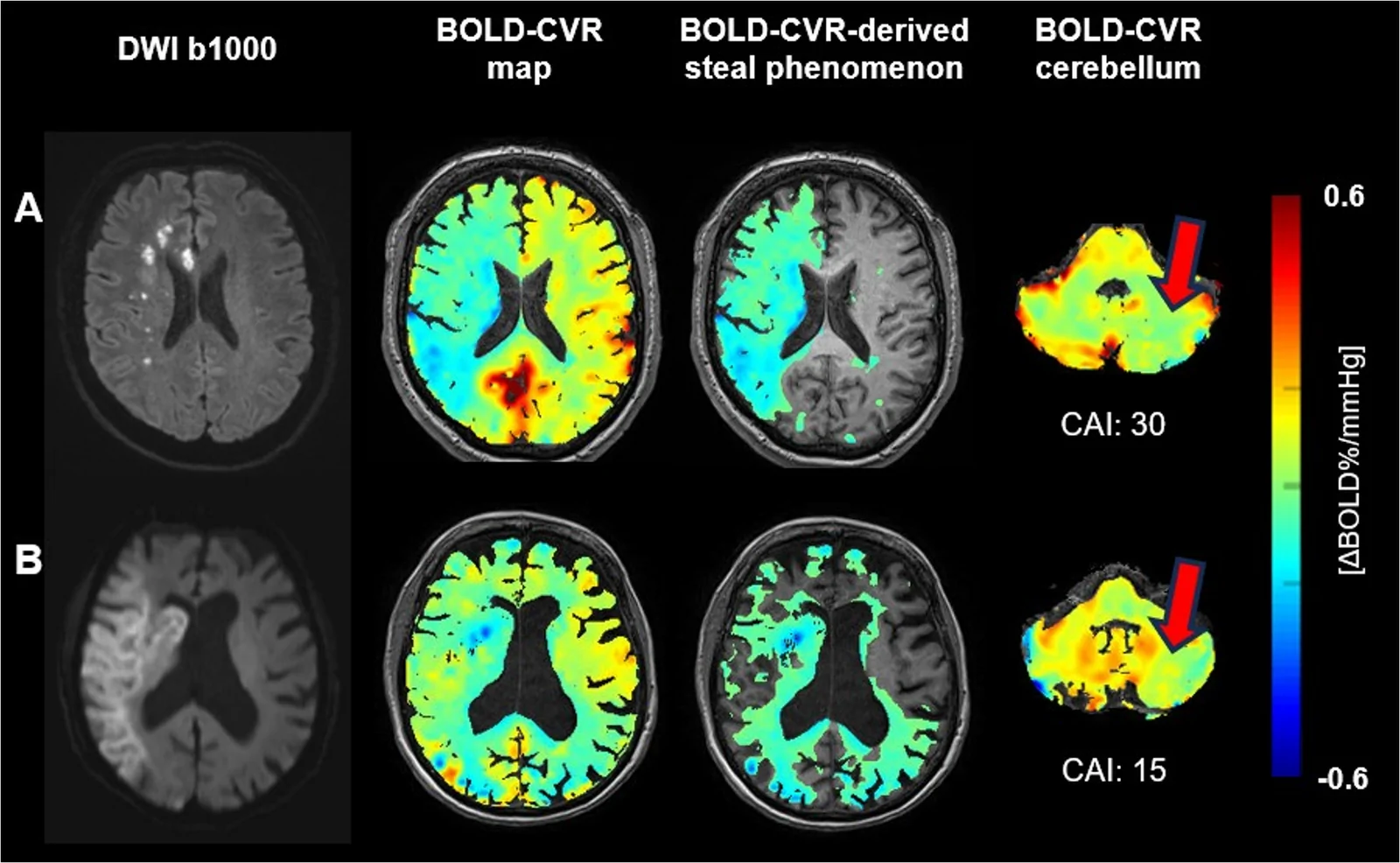
Illustrative examples of two patients. BOLD-CVR and trace DWI maps of two illustrative examples are presented. For each patient a whole-brain BOLD-CVR map, a BOLD-CVR map showing only steal phenomenon tissue areas, and trace DWI map is depicted. A, male subject (age range: 45–50 years) presented with ICA occlusion right and showed multiple, small infarct lesions in the ipsilateral MCA and ACA vascular territory lesion on DWI as well as extended supratentorial steal phenomenon and CCD on BOLD-CVR imaging. B, female subject (age range: 80–85 years) presented initially with ICA occlusion right and showed large infarct lesion in the whole ipsilateral MCA vascular territory on DWI as well as CCD on BOLD-CVR imaging after successful EVT. BOLD-CVR, blood oxygenation-level dependent cerebrovascular reactivity; CAI, cerebellar asymmetry index; DWI, diffusion-weighted imaging

In contrast, in the EVT group, CCD was associated with larger infarct volumes (64 [27–91] vs. 16 [5–28] cm^3^; *p* = 0.010), whereas BOLD-CVR values and steal phenomenon volumes did not differ between CCD-positive and CCD-negative patients (**Supplemental Table ****1**). Infarct volume remained independently associated with CCD in multivariable analysis (adjusted OR 5.75, 95% CI 1.41–68.92; *p* = 0.038; Table 2). Illustrative cases are shown in Fig. 3.

**Fig. 4:**
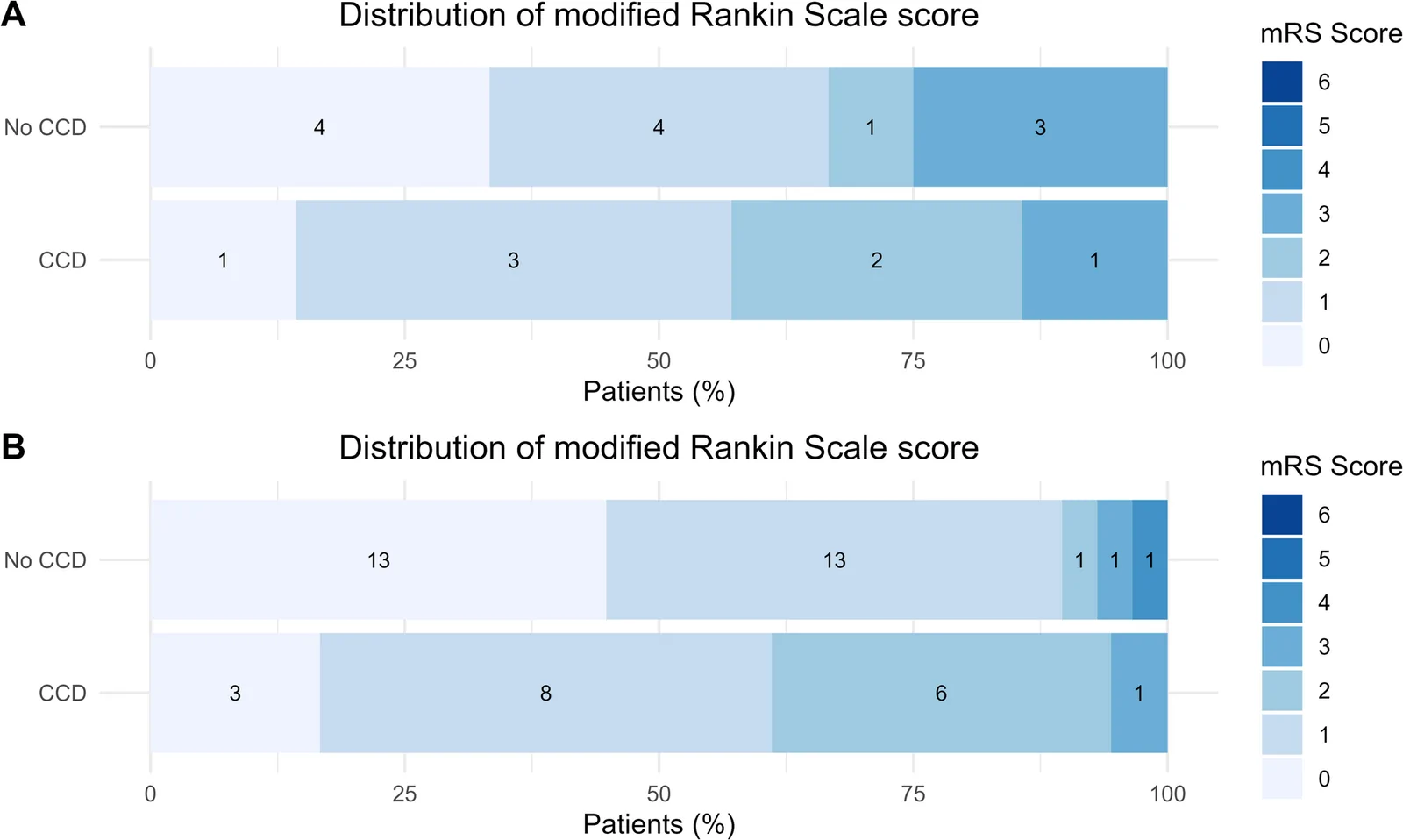
Distribution of 90-day modified Rankin Scale scores according to the presence of CCD. In panels A and B, the mRS score distributions for the EVT group (**A**) and non-EVT group (**B**), respectively, are shown. CCD, crossed cerebellar diaschisis; mRS, modified Rankin Scale

Median [IQR] 90-day mRS scores were 2 [2–3] in the EVT group and 2 [1–2] in the non-EVT group (Fig. 4). In complete-case analysis, CCD was independently associated with worse 90-day functional outcome in the non-EVT group (adjusted OR 6.59, 95% CI 1.47–31.70; *p* = 0.024), but not in the EVT group (Table 3). This association was attenuated and no longer statistically significant in sensitivity analyses using multiple imputation and best- and worst-case scenarios (**Supplemental Tables ****2****–****4**).

**Table 3:** Regression analysis of the association between CCD and 90-day functional outcome. Steal phenomenon volume encompasses the total volumes found within ACA and MCA vascular territories after subtracting the part included in the DWI infarct lesion. Steal phenomenon and infarct lesion size were scaled prior to regression model fitting. Regression analysis was performed separately in the EVT and non-EVT groups using ordinal logistic regression in a complete-case framework with 1’000-sample nonparametric bootstrap resampling and adjusted for infarct volume, steal volume, pre-stroke mRS, age, and group-specific covariates (TICI grade in the EVT group; bypass status in the non-EVT group). BOLD-CVR indicates blood oxygenation-level dependent cerebrovascular reactivity; CCD, crossed cerebellar diaschisis; CI, confidence interval; DWI, diffusion-weighted imaging; EC-IC, extra-to-intracranial; EVT, endovascular thrombectomy; mRS, modified Rankin Scale; OR, odds ratio. *P value < 0.05

	Independent variable	Univariable OR (95% CI)	*P* value	Multivariable OR (95% CI)	*P* value
EVT Group	Presence of CCD				
	No	**-**	0.63	**-**	0.88
	Yes	1.52 (0.29 to 8.30)		0.83 (0.07 to 9.26)	
	Steal phenomenon volume [cm^3^]			3.67 (0.80 to 21.54)	0.11
	DWI-derived infarct lesion volume [cm^3^]			1.44 (0.31 to 7.16)	0.63
	Pre-stroke mRS			1.45 (0.53 to 4.40)	0.47
	Age at ischemic event			0.94 (0.86 to 1.02)	0.17
	mTICI score				
	2b			-	
	2c			0.38 (0.01 to 32.90)	0.65
	3			1.77 (0.11 to 33.95)	0.68
Non-EVT Group	Presence of CCD				
	No	-	0.019*	-	0.024*
	Yes	4.17 (1.32 to 14.57)		6.59 (1.47 to 31.70)	
	Steal phenomenon volume [cm^3^]			1.36 (0.40 to 3.81)	0.75
	DWI-derived infarct lesion volume [cm^3^]			1.32 (0.52 to 2.84)	077
	Pre-stroke mRS			1.79 (0.76 to 3.96)	0.13
	Age at ischemic event			0.93 (0.85 to 1.04)	0.24
	EC-IC bypass surgery during follow-up time				
	No			-	0.52
	Yes			0.20 (0.010 to 3.88)	

## Discussion

This study investigated the occurrence of CCD in acute unilateral LVO-AIS in relation to two imaging markers – DWI-derived infarct lesion size and BOLD-CVR-derived steal phenomenon volume – while explicitly distinguishing patients imaged under persistent occlusion (non-EVT group) from those imaged after successful reperfusion (EVT group). This a priori stratification enabled exploration of two hypothesized and potentially coexisting CCD mechanisms: a functional, hemodynamic-induced CCD driven by impaired cerebrovascular reserve under persistent occlusion, and a structural, lesion-induced CCD related to irreversible supratentorial tissue damage after infarction.

Consistent with this conceptual framework, CCD in EVT-ineligible patients with persistent occlusion was independently associated with the extent of the steal phenomenon, whereas in successfully reperfused patients CCD was linked to larger infarct volumes.^7^–^12^ Overall CCD prevalence (35% in the EVT group and 41% in the non-EVT group) was comparable to prior reports^10^,^11^, confirming that CCD is common in the acute phase after ischemic stroke.

However, some important considerations should be mentioned. First, the present findings are based on observational associations, and causality cannot be inferred. The observed relationships between CCD, infarct volume, and hemodynamic impairment may be influenced by unmeasured factors. Second, the EVT and non-EVT groups represent fundamentally different clinical populations, with distinct stroke severity, infarct burden, and treatment pathways, and should therefore be interpreted as separate clinical phenotypes rather than directly comparable cohorts. Third, given the relatively small sample size, particularly in the EVT group, all findings should be interpreted with caution. The limited number of events results in reduced statistical power and wide confidence intervals, and therefore the observed associations should be considered exploratory and hypothesis-generating.

### CCD in the EVT Group

In the EVT group, CCD was associated with larger infarct volumes, supporting the concept of lesion-induced diaschisis through disruption of cortico-ponto-cerebellar pathways. This is anatomically plausible and aligns with longitudinal studies showing persistence of CCD after reperfusion in patients with large infarcts or incomplete tissue recovery.^8^.

However, the relationship between infarct volume and CCD is not uniform across literature. Several recent perfusion-based studies did not observe an association between infarct size and CCD.^10^–^12^ These differences likely reflect methodological variation, particularly imaging timing. In our cohort, imaging was performed after confirmed recanalization and when infarct demarcation was established, reducing confounding from transient hypoperfusion and acute treatment.

In contrast to some previous reports^8^,^19^, we did not observe an independent association between CCD and 90-day functional outcome in the EVT group, nor between infarct size and outcome, most likely reflecting limited statistical power due to the modest EVT sample size.

### CCD in the Non-EVT Group

The non-EVT group consisted of patients who were not eligible for recanalization therapies based on contemporary acute stroke guidelines after exclusion of patients with large infarct lesion.^20^ As expected, they had lower admission stroke severity and smaller infarcts. The presence of CCD was associated with hemodynamic impairment as quantified by steal phenomenon volume, whereas infarct lesion size showed no independent association. These findings align with perfusion-based CT, MRI, and PET/SPECT studies demonstrating that diaschisis severity scales with upstream hypoperfusion.^9^–^11^,^21^ Our results support the concept of predominantly hemodynamic-induced, potentially reversible cortico-cerebellar deafferentation in the absence of reperfusion.^8^,^9^,^22^.

Additionally, the presence of CCD in the non-EVT group was associated with poorer 90-day outcomes in the complete-case analysis, consistent with prior reports linking hemodynamic CCD to worse recovery^2^,^12^. Although this association was attenuated in sensitivity analyses accounting for missing data (multiple imputation, best-case and worst-case scenarios), the consistent direction of effect suggests potential clinical relevance that warrants confirmation in larger cohorts.

### Clinical Significance of CCD

Taken together, our findings may indicate that CCD reflects distinct underlying mechanisms depending on vascular status at imaging. Under persistent occlusion, CCD primarily reflects hemodynamic impairment and may identify patients at risk for early neurological deterioration and recurrent ischemia who could benefit from advanced or delayed revascularization strategies.^17^,^23^ After reperfusion, CCD appears to reflect structural injury rather than active hemodynamic impairment. Recognizing these mechanistic differences may refine imaging interpretation, improve prognostication, and support individualized treatment strategies in LVO-AIS. However, the clinical translation of BOLD-CVR imaging remains limited, as it requires specialized equipment for controlled CO_2_ delivery, standardized acquisition protocols, and dedicated post-processing, which are not widely available in routine acute stroke care and may restrict its broader implementation.

### Strength and Limitations

Our findings underscore the value of BOLD-CVR imaging for assessing CCD in LVO-AIS. Conceptually, BOLD-CVR-based assessment of CCD differs from perfusion- and metabolism-based techniques such as CT/MR perfusion and PET/SPECT. While PET/SPECT provides direct measures of cerebral blood flow and metabolism, BOLD-CVR reflects vascular reactivity and indirectly captures metabolic demand through neurovascular coupling. Thus, CCD is identified as an altered vascular response rather than a direct perfusion or metabolic deficit. In addition, BOLD-CVR assesses dynamic cerebrovascular reserve and steal phenomenon physiology in the supratentorial territories, complementing the static information provided by conventional perfusion imaging. By studying two well-defined populations – patients after successful EVT and EVT-ineligible patients with persistent occlusion – we were able to explore distinct pathophysiological mechanisms underlying CCD across different hemodynamic contexts.

However, some limitations must be acknowledged. First, the relatively small sample size – particularly in the EVT subgroup – limits generalizability, reduces statistical power, and results in wide confidence intervals, thereby limiting the precision of effect estimates. Consequently, study findings should be interpreted as exploratory and require confirmation in larger cohorts. Second, despite the use of multivariable analyses, differences in clinical characteristics and patient selection between the EVT and non-EVT groups may confound the observed associations, and the relative contributions of vascular status, stroke severity, and infarct burden cannot be fully disentangled. Third, study period spanned nearly nine years (2015–2024), during which EVT techniques, devices, and selection criteria evolved, potentially introducing heterogeneity within the EVT group. Fourth, the COVID-19 pandemic fell within the study period and may have affected patient presentation patterns, resource availability, and treatment timing. Furthermore, BOLD-CVR imaging was performed up to 7 days after stroke onset, which may introduce variability in CCD expression given its dynamic evolution. To account for this, time from symptom onset to imaging was included as a covariate in multivariable analyses. Additionally, the CAI threshold used to define CCD was derived from previous studies and has not been validated in acute stroke patients. Although this approach ensures comparability with prior studies, the optimal threshold for defining CCD in acute ischemic stroke remains to be established. In the non-EVT group, EC-IC bypass surgery was included as a covariate in outcome models to account for its potential confounding effect. However, as this intervention may lie on the causal pathway related to hemodynamic impairment residual confounding may still be present, and results should be interpreted accordingly. Lastly, pre-treatment BOLD-CVR imaging was not feasible due to clinical urgency and ethical constraints, limiting direct comparison with hyperacute CCD assessments reported previously.^10^ The exclusion of patients with large infarcts in the non-EVT group, based on predefined study criteria, may introduce selection bias.

## Conclusions

In acute LVO-AIS, CCD detected by BOLD-CVR reflects different underlying mechanisms depending on vascular status at the time of imaging. Under persistent occlusion, CCD is mainly driven by hemodynamic impairment – particularly the steal phenomenon – whereas after successful reperfusion, CCD more closely reflects structural ischemic injury. BOLD-CVR imaging provides valuable mechanistic insight into CCD, but larger studies are required to validate these findings and clarify the clinical significance of CCD in acute ischemic stroke care.

## Supplementary Information

Below is the link to the electronic supplementary material.

Supplementary Material 1

## Data Availability

In addition to the detailed methodological description provided in this report, numeric data of the included patients can also be made available in anonymized format. Please contact the corresponding author for such a request.

## Abbreviations

ACA: Anterior cerebral artery
ADC: Apparent diffusion coefficient
AIS: Acute ischemic stroke
BOLD: Blood oxygenation-level dependent
CAI: Cerebellar asymmetry index
CD: Ccrossed cerebellar diaschisis
CT: Computed tomography
CVR: Cerebrovascular reactivity
DWI: Diffusion-weighted imaging
EVT: Endovascular treatment
LVO: Large-vessel occlusion
MCA: Middle cerebral artery
MRI: Magnetic resonance imaging
mRS: modified Rankin Scale
mTICI: modified Thrombolysis In Cerebral Infarction
NIHSS: National Institutes of Health Stroke Scale
PCA: Posterior cerebral artery
PET: Positron emission tomography
SPECT: Single photon emission computed tomography
