# Supplementary material for: Deciphering Crossed Cerebellar Diaschisis in Patients with Large-Vessel Occlusion Acute Ischemic Stroke using BOLD Cerebrovascular Reactivity
Source: Cerebellum. 2026 May 7;25(3):72. doi: 10.1007/s12311-026-02014-1 (PMC13649813; doi:10.1007/s12311-026-02014-1)
Supplement: Supplementary file 1 [file 12311_2026_2014_MOESM1_ESM.docx]

# SUPPLEMENTAL MATERIALS

**Supplemental Methods**

Inclusion and exclusion criteria

Inclusion criteria

1. Acute clinical ischemic stroke at hospital admission
2. First-ever LVO diagnosis of the anterior circulation, including occlusion of the internal carotid artery (ICA) and/or of M1-segment of the middle cerebral artery (MCA)
3. Maximal 7 days delay between completion of imaging examination and symptom stroke onset or, in case of unclear symptom onset time, LVO detection time
4. 18 years or above
5. Living independent before stroke (modified Ranking Scale, mRS ≤3)
6. Written informed consent of the patient or when the patient is not able to participate in the consenting procedure, the written authorization of an independent doctor who is not involved in the research project to safeguard the interests of the patients (in that case, post-hoc written informed consent of the patient or next of kin had to be obtained).

Exclusion criteria

1. Imaging signs of cerebellar infarction or other cerebellar pathology
2. Known chronic vessel occlusions
3. Nonthrombotic/embolic etiologies of the LVO such as Moyamoya syndrome or vasculitis
4. Major cardiac, psychiatric and/or neurological diseases
5. Early seizures
6. Known or suspected non-compliance, drug and/or alcohol abuse
7. For patients ineligible for EVT: infarct core volume >70 mL
8. Contra-indications for MRI
9. Documented evidence that the patient does not want to participate in any scientific study.

Ineligibility criteria for endovascular thrombectomy

In general, according to current guidelines^1^, patients were considered not eligible for EVT if they:

- Presented within 6 hours from ischemic symptom onset time with
  - NIHSS score <6 or ASPECTS <6
- Or presented between 6 and 24 hours from ischemic symptom onset time with
  - NIHSS score <6, infarct-core volume >70 mL, penumbra volume <15 mL or penumbra-core ratio <1.8

However, at our institution, patients with NIHSS <6 that present within 24h from symptom onset are treated in some selected situations. The decision to perform or not immediate EVT was evaluated case by case by both the senior neuroradiologist and neurologist on call.

Baseline characteristics

Demographic and clinical baseline characteristics were collected for each patient, encompassing stroke severity at hospitalization, symptom onset time, cerebrovascular risk factors (smoking history, dyslipidemia, obesity, diabetes mellitus, coronary artery disease, peripheral artery disease), history of atrial fibrillation, symptomatic side, and localization of the occluded vessel (ICA, M1-MCA, or both). Initial stroke severity, assessed by a certified physician upon arrival at our center, was determined using the National Institutes of Health Stroke Scale (NIHSS) and modified Rankin Scale (mRS) score. The diagnosis was categorized as either ischemic stroke or TIA, in accordance with the acute ischemic stroke definition set out by the American Heart Association.^2^

Acute stroke management

Usual care for patients with LVO-AIS who did or did not undergo EVT consisted of at least 24 hours of continuous monitoring and specialized care on a stroke unit. If after 24 hours patients were considered in a stable condition and not requiring intermediate care, they were transferred to post-stroke unit or regular ward care. In cases of symptom deterioration in patients who did not undergo EVT, blood pressure was maintained at permissive hypertensive values (i.e., systolic 160-180 mmHg) and the indication for rescue extra-to-intracranial flow augmentation bypass surgery was made based on interdisciplinary expert consensus.^3^

BOLD-CVR imaging protocol

BOLD-CVR was acquired using an EPI sequence (TR = 2000 ms, TE = 30 ms, flip angle = 85°, 35 slices, slice thickness = 3.0 mm, FOV = 192 × 192 mm2, matrix = 64 × 64, voxel size ≈ 3.0 × 3.0 × 3.0 mm3, 200 dynamics, interleaved acquisition, motion correction and spatial filtering applied). DWI was performed with a single-shot EPI sequence (TR = 2000 ms, TE = 30 ms, slice thickness = 3.0 mm, FOV = 192 × 192 mm2). High-resolution T1-weighted anatomical imaging (MPRAGE) was acquired with TR = 2200 ms, TE = 5.17 ms, TI = 900 ms, flip angle = 8°, 176 slices, slice thickness = 1.0 mm, FOV = 230 × 230 mm2, GRAPPA factor = 2, voxel size ≈ 0.8 × 0.8 × 1.0 mm3.

During BOLD-CVR acquisition, a standardized carbon dioxide (CO₂) stimulus was administered using the RespirAct™ system (Thornhill Research Institute, Toronto, Canada), which allows precise targeting of end-tidal CO₂ pressure (PetCO₂) while maintaining isoxia (constant oxygen levels)^4^. The stimulus protocol consisted of 100 seconds at the patient-specific baseline PetCO₂, followed by a 10mmHg increase sustained for 80 seconds, and a return to baseline for an additional 120 seconds. End-tidal oxygen pressure (PetO₂) was maintained at the patient-specific resting value throughout the protocol. All imaging was acquired in a single session. Image quality was rigorously assessed, and examinations were excluded in cases of excessive head motion (frame-wise displacement >2 mm) or insufficient CO₂ stimulus amplitude (<6 mmHg PetCO₂ step change). Vital signs (including blood pressure, oxygen saturation, and electrocardiogram) were continuously monitored during the session to promptly address any potential adverse events. Moreover, NIHSS score was evaluated by a physician both before and after the BOLD-CVR scan to provide insights into the patients' neurological condition and any changes possibly induced by the imaging procedure.

Image processing

BOLD-CVR values, representing the percent change in BOLD signal per mmHg change in end-tidal CO_2_ (PetCO_2_) during the hypercapnic stimulus, were computed using a previously validated analysis pipeline.^5^ After imaging data preprocessing – which included slice-time correction, motion realignment, spatial smoothing with a Gaussian kernel, and temporal filtering – subject-space BOLD-CVR maps were generated by calculating the voxelwise percentage signal change per mmHg PetCO_2_ (%ΔBOLD/mmHg) using MATLAB 2022 (The MathWorks, Inc.) and SPM12 (Wellcome Trust Centre for Neuroimaging, UCL). The resulting maps were co-registered to each subject’s anatomical T1-weighted image using rigid-body transformation. Quantitative BOLD-CVR values were extracted from predefined regions of interest (ROIs), including ipsilateral and contralateral hemispheres, gray and white matter, and vascular territories (ACA, MCA, and PCA), as defined by the vascular atlases described by Liu et al.^6^ Brain regions exhibiting hemodynamic impairment (i.e., steal phenomenon) were identified and quantified following an established method.^7,8^ Steal regions were defined as voxels showing negative BOLD-CVR values (i.e., <0% ΔBOLD/mmHg) after exclusion of those within the infarct lesion mask. Unless otherwise specified, reported steal phenomenon volumes refer to the total volume of negatively reactive tissue within the affected ACA and MCA vascular territories. The cerebellar asymmetry index (CAI) was calculated using a predefined cerebellar ROI as previously described.^5,9^ CAI is defined as:

$$CAI= \frac{{meanBOLD-CVR}_{ipsilateral}- {meanBOLD-CVR}_{contralateral}}{{meanBOLD-CVR}_{ipsilateral}}$$

where the ipsilateral hemisphere refers to the cerebellar side corresponding to the supratentorial ischemic lesion.^5^

Ischemic infarct lesions were automatically segmented from diffusion-weighted images (b_0_, b_1000_, and apparent diffusion coefficient maps) using a deep learning-based algorithm developed by Liu et al.^10^, trained and tested in 2628 brain MRIs and publicly available at <https://www.nitrc.org/projects/ads>. Segmented lesion masks were co-registered to each subject’s anatomical T1 space using rigid-body transformation. To avoid confounding from irreversibly damaged tissue, voxels within the infarct mask were excluded from all ROI calculations of mean BOLD-CVR values. Unless stated otherwise, all reported BOLD-CVR metrics represent mean values excluding infarcted voxels.

Imaging acquisition parameters

TA: 6:46 min Coil Selection: Auto Voxel Size: 3.0×3.0×3.0 mm³ Acc:: 2 Rel. SNR: 1.00

**\\USER\test\test\CRPPstroke20191228_32ch_New_Patients6-dwicvrt1\CO2_bold ***

Properties

Start measurement without further	On preparation

Wait for User to Start	Off

Start measurements	Single Measurement

Prio Recon	Off

Auto Open Inline Display	Off

Auto Close Inline Display	Off

Load Images to MR View&GO	On

Auto Store Images	On Load Images to Stamp Segments	Off Load Images to Graphic Segments	Off Graphic segment	Default

Inline Movie	Off

Routine

| Slice Group | 1 |
| --- | --- |
| Slices | 35 |
| Distance Factor | 10 % |
| Position | R0.8 P5.9 H10.6 mm |
| Orientation | T > C-16.5 > S0.6 |
| Phase Encoding Dir. | A >> P |
| Phase Oversampling | 0 % |
| FoV Read | 192 mm |
| FoV Phase | 100.0 % |
| Slice Thickness | 3.0 mm |
| TR | 2000.0 ms |
| TE | 30.00 ms |
| Averages | 1 |
| Concatenations | 1 |
| AutoAlign | Head > Brain |
| Coil Elements | HEA;HEP |

Contrast - Common

TR	2000.0 ms

TE	30.00 ms

MTC	Off

Flip Angle	85 deg

Fat-Water Contrast	Fat Saturation

Reconstruction	Magnitude

Contrast - Dynamic

Dynamic Mode	Standard

Measurements	200

Delay in TR	0.00 ms

Resolution - Common

FoV Read	192 mm

FoV Phase	100.0 %

Slice Thickness	3.0 mm

Base Resolution	64

Phase Resolution	100 %

Interpolation	Off

Resolution - Acceleration

Acceleration mode	GRAPPA

Reference Scans	EPI/Separate

Acceleration Factor PE	2

Reference Lines PE	32

Phase Partial Fourier	Off

Resolution - Filter

Raw Filter	Off

Elliptical Filter	On

Hamming	Off

Distortion Correction	Off

Normalize	Prescan

Geometry - Common

| Slice Group | 1 |
| --- | --- |
| Slices | 35 |
| Distance Factor | 10 % |
| Position | R0.8 P5.9 H10.6 mm |
| Orientation | T > C-16.5 > S0.6 |
| Phase Encoding Dir. | A >> P |
| Phase Oversampling | 0 % |
| FoV Read | 192 mm |
| FoV Phase | 100.0 % |
| Slice Thickness | 3.0 mm |
| TR | 2000.0 ms |
| Multi-Slice Mode | Interleaved |
| Series | Interleaved |
| Concatenations | 1 |

Geometry - AutoAlign

| Slice Group | 1 |
| --- | --- |
| Position Orientation  Phase Encoding Dir. | R0.8 P5.9 H10.6 mm T > C-16.5 > S0.6  A >> P |
| AutoAlign | Head > Brain |
| Initial Position | R0.8 P5.9 H10.6 |
| R | 0.8 mm |
| P | 5.9 mm |
| H | 10.6 mm |
| Initial Orientation | T > C |
| T > C | -16.50 |
| > S | 0.60 |
| Initial Rotation | -1.12 deg |

Geometry - Saturation

Special Saturation	None

Geometry - Tim Planning Suite

Set-n-Go Protocol	Off

Table Position	0 mm

Table Position	H

Inline Composing	Off

System - Miscellaneous

Coil Selection	Auto Coil Select

MSMA	S - C - T

Sagittal	R >> L

Coronal	A >> P

Transversal	F >> H

Coil Combination	Adaptive Combine

Matrix Optimization	Off

System - Adjustments

Adjustment Strategy	Standard

B0 Shim	Standard

B1 Shim	TrueForm

Adjustment Tolerance	Auto

System - Adjustments

Adjust with Body Coil	Off

Confirm Frequency	Never

Assume Silicone	Off

System - Adjust Volume

Position	R0.8 P5.9 H10.6 mm

Orientation	T > C-16.5 > S0.6

Rotation	-1.12 deg

A >> P	192 mm

R >> L	192 mm

F >> H	116 mm

Reset	Off

System - Tx/Rx

Frequency 1H	123.256368 MHz

? Ref. Amplitude 1H	0.000 V

Reset	Off

Correction Factor	1.00

Image Scaling	1.000

Physio - Signal

1st Signal/Mode	None

TR	2000.0 ms

Concatenations	1

BOLD

GLM Statistics	Off

Ignore Meas. at Start	0

Ignore After Transition	0

Model Transition States	On

Temp. Highpass Filter	On

Threshold	2.50

Paradigm Size	66

Meas[1]	Active

Meas[2]	Active

Meas[3]	Active

Meas[4]	Active

Meas[5]	Active

Meas[6]	Active

Meas[7]	Active

Meas[8]	Active

Meas[9]	Active

Meas[10]	Active

Meas[11]	Active

Meas[12]	Active

Meas[13]	Active

Meas[14]	Active

Meas[15]	Active

Meas[16]	Active

Meas[17]	Active

Meas[18]	Active

Meas[19]	Active

Meas[20]	Active

Meas[21]	Active

Meas[22]	Active

Meas[23]	Ignore

Meas[24]	Ignore

Meas[25]	Ignore

Meas[26]	Ignore

Meas[27]	Ignore

Meas[28]	Ignore

Meas[29]	Ignore

Meas[30]	Ignore

Meas[31]	Ignore

BOLD

Meas[32]	Ignore

Meas[33]	Ignore

Meas[34]	Ignore

Meas[35]	Ignore

Meas[36]	Ignore

Meas[37]	Ignore

Meas[38]	Ignore

Meas[39]	Ignore

Meas[40]	Ignore

Meas[41]	Ignore

Meas[42]	Ignore

Meas[43]	Ignore

Meas[44]	Ignore

Meas[45]	Active

Meas[46]	Active

Meas[47]	Active

Meas[48]	Active

Meas[49]	Active

Meas[50]	Active

Meas[51]	Active

Meas[52]	Active

Meas[53]	Active

Meas[54]	Active

Meas[55]	Active

Meas[56]	Active

Meas[57]	Active

Meas[58]	Active

Meas[59]	Active

Meas[60]	Active

Meas[61]	Active

Meas[62]	Active

Meas[63]	Active

Meas[64]	Active

Meas[65]	Active

Meas[66]	Active

Motion Correction	On

Spatial Filter	On

Filter Width	4.0 mm

Measurements	200

Delay in TR	0.00 ms

Sequence - Part 1

Sequence Name	epfid

Excitation	Standard

RF Pulse Type	Normal

Gradient Mode	Fast*

Bandwidth	2368 Hz/Px

Echo Spacing	0.53 ms

Free Echo Spacing	Off

EPI Factor	64

Sequence - Part 2

Introduction	Off

TA: 8:14 min Coil Selection: Auto Voxel Size: 0.8×0.8×1.0 mm³ Acc:: 2 Rel. SNR: 1.00

**\\USER\test\test\CRPPstroke20191228_32ch_New_Patients6-dwicvrt1\t1mprage_tra_HighRes_Nch_ 32ch ***

Properties

Start measurement without further	On preparation

Wait for User to Start	Off

Start measurements	Single Measurement

Prio Recon	Off

Auto Open Inline Display	Off

Auto Close Inline Display	Off

Load Images to MR View&GO	On

Auto Store Images	On Load Images to Stamp Segments	Off Load Images to Graphic Segments	Off Graphic segment	Default

Inline Movie	Off

Routine

| Slab Group | 1 | |
| --- | --- | --- |
| Slabs | 1 |  |
| Distance Factor | 50 % |  |
| Position | L0.0 P1.6 H13.6 | mm |
| Orientation | Transversal |  |
| Phase Encoding Dir. | R >> L |  |
| Slices per Slab | 176 | |
| Phase Oversampling | 10 % | |
| Slice Oversampling | 27.3 % | |
| FoV Read | 230 mm | |
| FoV Phase | 100.0 % | |
| Slice Thickness | 1.0 mm | |
| TR | 2200.0 ms | |
| TE | 5.17 ms | |
| Averages | 1 | |
| Concatenations | 1 | |
| AutoAlign | Head > Brain | |
| Coil Elements | HEA;HEP | |

Contrast - Common

TR	2200.0 ms

TE	5.17 ms

Magn. Preparation	Non-sel. IR

TI	900 ms

Flip Angle	8 deg

Fat-Water Contrast	Standard

Dark Blood	Off

Reconstruction	Magnitude

Contrast - Dynamic

Dynamic Mode	Standard

Measurements	1

Multiple Series	Each Measurement

Reordering	Linear Rot.

Resolution - Common

FoV Read	230 mm

FoV Phase	100.0 %

Slice Thickness	1.0 mm

Base Resolution	288

Phase Resolution	100 %

Slice Resolution	100 %

Interpolation	Off

Resolution - Acceleration

Acceleration mode	GRAPPA

Reference Scans	Integrated

Acceleration Factor PE	2

Reference Lines PE	24

Acceleration Factor 3D	1

Phase Partial Fourier	Off

Slice Partial Fourier	Off

Asymmetric Echo	Allowed

Elliptical Scanning	Off

Resolution - Filter

Raw Filter	Off

Elliptical Filter	Off

Distortion Correction	3D

Normalize	Prescan

Image Filter	On

Geometry - Common

| Slab Group | 1 |
| --- | --- |
| Slabs | 1 |
| Distance Factor | 50 % |
| Position | L0.0 P1.6 H13.6 mm |
| Orientation | Transversal |
| Phase Encoding Dir. | R >> L |
| Slices per Slab | 176 |
| Phase Oversampling | 10 % |
| Slice Oversampling | 27.3 % |
| FoV Read | 230 mm |
| FoV Phase | 100.0 % |
| Slice Thickness | 1.0 mm |
| TR | 2200.0 ms |
| Multi-Slice Mode | Single Shot |
| Series | Ascending |
| Concatenations | 1 |

Geometry - AutoAlign

| Slab Group | 1 |
| --- | --- |
| Position Orientation  Phase Encoding Dir. | L0.0 P1.6 H13.6 mm  Transversal R >> L |
| AutoAlign | Head > Brain |
| Initial Position | L0.0 P1.6 H13.6 |
| R | 0.0 mm |
| P | 1.6 mm |
| H | 13.6 mm |
| Initial Orientation | Transversal |
| Initial Rotation | 91.66 deg |

Geometry - Navigator Geometry - Tim Planning Suite

Set-n-Go Protocol	Off

Table Position	0 mm

Table Position	H

Inline Composing	Off

System - Miscellaneous

Coil Selection	Auto Coil Select

MSMA	S - C - T

System - Miscellaneous

Sagittal	R >> L

Coronal	A >> P

Transversal	F >> H

Coil Combination	Adaptive Combine

Matrix Optimization	Off

System - Adjustments

Adjustment Strategy	Standard

B0 Shim	Tune up

B1 Shim	TrueForm

Adjustment Tolerance	Auto

Adjust with Body Coil	Off

Confirm Frequency	Never

Assume Silicone	Off

System - Adjust Volume

Position	Isocenter

Orientation	Transversal

Rotation	0.00 deg

A >> P	263 mm

R >> L	350 mm

F >> H	350 mm

Reset	Off

System - Tx/Rx

Frequency 1H	123.256368 MHz

? Ref. Amplitude 1H	0.000 V

Reset	Off

Correction Factor	1.00

Image Scaling	1.000

Physio - Signal

1st Signal/Mode	None

TR	2200.0 ms

Concatenations	1

Physio - Cardiac

Fat-Water Contrast	Standard

Magn. Preparation	Non-sel. IR

TI	900 ms

Dark Blood	Off

FoV Read	230 mm

FoV Phase	100.0 %

Phase Resolution	100 %

Dynamic Mode	Standard

Physio - PACE

Resp. Control	Off

Concatenations	1

Inline - Subtraction

Subtract	Off

Measurements	1

StdDev	Off

Save Original Images	On

Inline - Cardiac

Inline - MIP

MIP Sag	Off

MIP Cor	Off

MIP Tra	Off

MIP Time	Off

Radial MIP	Off

Save Original Images	On

MPR Sag	Off

MPR Cor	Off

MPR Tra	Off

Inline - Composing

Inline Composing	Off

Sequence - Part 1

Sequence Name	tfl_r

Dimension	3D

Excitation	Slab-sel.

RF Pulse Type	Fast

Gradient Mode	Normal

Flow Compensation	On

Reordering	Linear Rot.

Bandwidth	250 Hz/Px

Echo Spacing	10.18 ms

Asymmetric Echo	Allowed

Turbo Factor	317

Sequence - Part 2

Introduction	On

RF Spoiling	On

Incr. Gradient Spoiling	On

Sequence - Assistant

SAR Assistant	Off

Magn. Preparation	Non-sel. IR

Save Original Images	On

TE	5.17 ms

TR	2200.0 ms

**Supplemental Tables**

| **Imaging parameters** | **EVT Group (n=23)** | | | **No-EVT Group (n=56)** | | | | |
| --- | --- | --- | --- | --- | --- | --- | --- | --- |
|  | **CCD (n=8)** | **No CCD (n=15)** | ***P* value^†^** | **CCD (n=23)** | **No CCD (n=33)** | | ***P* value^†^** | |
| **Morphological findings** |  |  |  |  | |  | |  |
| DWI-derived infarct lesion size [cm^3^]  (median [IQR]) | 63.7 [27.2, 91.5] | 16.2 [4.7, 28.5] | **0.010*** | 6.2 [0.6, 14.9] | | 0.8 [0.0, 2.3] | | **0.009*** |
| **CAI** |  |  |  |  | |  | |  |
| Median [IQR] | -4.7 [-14.1, 3.6] | 19.4 [14.2, 28.0] | **<0.001*** | 24.3 [15.6, 38.9] | | 1.4 [-7.9, 4.1] | | **<0.001*** |
| **Hemodynamic findings** |  |  |  |  | |  | |  |
| BOLD-CVR values in MCA and ACA territory  [% ΔBOLD/mmHg]  (mean (SD)) | 0.10 (0.07) | 0.10 (0.04) | 0.84 | 0.00 (0.08) | | 0.06 (0.06) | | **0.003*** |
| Steal phenomenon volume in MCA and ACA territory [cm^3^] (median [IQR])^#^ | 25.5 [10.6, 52.2] | 13.5 [5.1, 31.5] | 0.30 | 178.7 [105.3, 297.9] | | 65.4 [22.7, 174.9] | | **0.002*** |

**Supplemental Table 1. Morphological and hemodynamic imaging findings.**

ACA, anterior cerebral artery; CAI, cerebellar asymmetry index; CCD, crossed cerebellar diaschisis; EC-IC, extra-to-intracranial; ICA, internal carotid artery; IQR, interquantile range; MCA, middle cerebral artery; NIHSS, National Institutes of Health Stroke Scale; mRS, modified Ranking Scale; TIA, transient ischemic attack. **^†^***P* values are exploratory in nature and should be interpreted accordingly. Between-group comparisons were conducted using the Pearson χ^2^ or Fisher exact test for categorical variables, and Student t test or Mann-Whitney U test for continuous variables respectively, as appropriate based on normality testing. ^#^The part of steal phenomenon included in the DWI-derived infarct lesion was subtracted from the total amount. **P* value < 0.05.

|  | **Independent Variable** | **Univariable OR**  **(95% CI)** | ***P* value** | **Multivariable OR**  **(95% CI)** | ***P* value** |
| --- | --- | --- | --- | --- | --- |
| **EVT Group** | Presence of CCD |  |  |  |  |
|  | No | **-** | 0.54 | **-** | 0.84 |
|  | Yes | 1.66 (0.29 to 9.54) |  | 1.26 (0.10 to 16.67) |  |
|  | Steal phenomenon volume [cm^3^] |  |  | 4.16 (0.64 to 26.79) | 0.11 |
|  | DWI-derived infarct lesion volume [cm^3^] |  |  | 1.11 (0.19 to 6.52) | 0.89 |
|  | Pre-stroke mRS |  |  | 1.07 (0.31 to 3.61) | 0.89 |
|  | Age at ischemic event |  |  | 0.96 (0.88 to 1.05) | 0.34 |
|  | mTICI score |  |  |  |  |
|  | 2b |  |  | - |  |
|  | 2c |  |  | 3.10 (0.01 to 30.91) | 0.58 |
|  | 3 |  |  | 1.29 (0.03 to 50.70) | 0.87 |
| **Non-EVT Group** | Presence of CCD |  |  |  |  |
|  | No | - | **0.034*** | - | 0.090 |
|  | Yes | 4.02 (1.11 to 14.51) |  | 3.65 (0.80 to 16.70) |  |
|  | Steal phenomenon volume [cm^3^] |  |  | 1.15 (0.51 to 2.58) | 0.72 |
|  | DWI-derived infarct lesion volume [cm^3^] |  |  | 1.24 (0.65 to 2.36) | 0.50 |
|  | Pre-stroke mRS |  |  | 1.73 (1.01 to 2.96) | **0.045*** |
|  | Age at ischemic event |  |  | 0.97 (0.91 to 1.04) | 0.51 |
|  | EC-IC bypass surgery during follow-up time |  |  |  |  |
|  | No |  |  | - | 0.67 |
|  | Yes |  |  | 0.67 (0.10 to 4.48) |  |

**Supplemental Table 2. Regression analysis of the association between CCD and 90-day functional outcome after multiple imputation.**

Results of proportional-odds ordinal logistic regression models after handling missing outcomes using multiple imputation by chained equations (20 imputations). Regression analysis was performed separately in the EVT and non-EVT groups and adjusted for infarct volume, steal volume, pre-stroke mRS, age, and group-specific covariates (TICI grade in the EVT group; bypass status in the non-EVT group). BOLD-CVR indicates blood oxygenation-level dependent cerebrovascular reactivity; CCD, crossed cerebellar diaschisis; CI, confidence interval; DWI, diffusion-weighted imaging; EC-IC, extra-to-intracranial; EVT, endovascular thrombectomy; mRS, modified Rankin Scale; OR, odds ratio. **P* value < 0.05.

|  | **Independent Variable** | **Univariable OR**  **(95% CI)** | ***P* value** | **Multivariable OR**  **(95% CI)** | ***P* value** |
| --- | --- | --- | --- | --- | --- |
| **EVT Group** | Presence of CCD |  |  |  |  |
|  | No | **-** | 0.47 | **-** | 0.78 |
|  | Yes | 1.75 (0.37 to 8.45) |  | 1.33 (0.15 to 12.16) |  |
|  | Steal phenomenon volume [cm^3^] |  |  | 2.54 (0.76 to 10.12) | 0.14 |
|  | DWI-derived infarct lesion volume [cm^3^] |  |  | 0.94 (0.22 to 3.86) | 0.94 |
|  | Pre-stroke mRS |  |  | 1.55 (0.68 to 4.01) | 0.31 |
|  | Age at ischemic event |  |  | 0.91 (0.84 to 0.98) | **0.019*** |
|  | mTICI score |  |  |  |  |
|  | 2b |  |  | - |  |
|  | 2c |  |  | 0.83 (0.02 to 21.30) | 0.91 |
|  | 3 |  |  | 5.20 (0.58 to 33.95) | 0.15 |
| **Non-EVT Group** | Presence of CCD |  |  |  |  |
|  | No | - | **0.042*** | - | 0.084 |
|  | Yes | 3.00 (1.05 to 8.95) |  | 2.94 (0.87 to 10.42) |  |
|  | Steal phenomenon volume [cm^3^] |  |  | 1.17 (0.59 to 2.29) | 0.64 |
|  | DWI-derived infarct lesion volume [cm^3^] |  |  | 1.33 (0.71 to 2.80) | 0.38 |
|  | Pre-stroke mRS |  |  | 1.28 (0.82 to 2.00) | 0.26 |
|  | Age at ischemic event |  |  | 0.93 (0.87 to 0.99) | **0.045*** |
|  | EC-IC bypass surgery during follow-up time |  |  |  |  |
|  | No |  |  | - | 0.54 |
|  | Yes |  |  | 0.59 (0.10 to 3.14) |  |

**Supplemental Table 3. Regression analysis of the association between CCD and 90-day functional outcome in best-case scenario framework.**

Results of proportional-odds ordinal logistic regression models under the best-case assumption, in which all missing outcomes were assigned mRS = 0. Regression analysis was performed separately in the EVT and non-EVT groups and adjusted for infarct volume, steal volume, pre-stroke mRS, age, and group-specific covariates (TICI grade in the EVT group; bypass status in the non-EVT group). BOLD-CVR indicates blood oxygenation-level dependent cerebrovascular reactivity; CCD, crossed cerebellar diaschisis; CI, confidence interval; DWI, diffusion-weighted imaging; EC-IC, extra-to-intracranial; EVT, endovascular thrombectomy; mRS, modified Rankin Scale; OR, odds ratio. **P* value < 0.05.

|  | **Independent Variable** | **Univariable OR**  **(95% CI)** | ***P* value** | **Multivariable OR**  **(95% CI)** | ***P* value** |
| --- | --- | --- | --- | --- | --- |
| **EVT Group** | Presence of CCD |  |  |  |  |
|  | No | **-** | 0.94 | **-** | 0.50 |
|  | Yes | 1.05 (0.23 to 4.74) |  | 0.51 (0.06 to 3.64) |  |
|  | Steal phenomenon volume [cm^3^] |  |  | 1.74 (0.56 to 5.50) | 0.32 |
|  | DWI-derived infarct lesion volume [cm^3^] |  |  | 1.59 (0.39 to 6.93) | 0.51 |
|  | Pre-stroke mRS |  |  | 1.09 (0.51 to 2.40) | 0.80 |
|  | Age at ischemic event |  |  | 1.01 (0.95 to 1.08) | 0.59 |
|  | mTICI score |  |  |  |  |
|  | 2b |  |  | - |  |
|  | 2c |  |  | 2.87 (0.10 to 30.91) | 0.51 |
|  | 3 |  |  | 0.85 (0.10 to 7.00) | 0.88 |
| **Non-EVT Group** | Presence of CCD |  |  |  |  |
|  | No | - | **0.013*** | - | 0.061 |
|  | Yes | 3.75 (1.34 to 11.13) |  | 2.98 (0.95 to 9.66) |  |
|  | Steal phenomenon volume [cm^3^] |  |  | 1.40 (0.72 to 2.80) | 0.31 |
|  | DWI-derived infarct lesion volume [cm^3^] |  |  | 0.98 (0.52 to 1.78) | 0.94 |
|  | Pre-stroke mRS |  |  | 1.67 (1.10 to 2.60) | **0.016*** |
|  | Age at ischemic event |  |  | 0.99 (0.94 to 1.05) | 0.90 |
|  | EC-IC bypass surgery during follow-up time |  |  |  |  |
|  | No |  |  | - | 0.54 |
|  | Yes |  |  | 0.77 (0.15 to 3.99) |  |

**Supplemental Table 4. Regression analysis of the association between CCD and 90-day functional outcome in worst-case scenario framework.**

Results proportional-odds ordinal logistic regression models under the worst-case assumption, in which all missing outcomes were assigned mRS = 6. Regression analysis was performed separately in the EVT and non-EVT groups and adjusted for infarct volume, steal volume, pre-stroke mRS, age, and group-specific covariates (TICI grade in the EVT group; bypass status in the non-EVT group). BOLD-CVR indicates blood oxygenation-level dependent cerebrovascular reactivity; CCD, crossed cerebellar diaschisis; CI, confidence interval; DWI, diffusion-weighted imaging; EC-IC, extra-to-intracranial; EVT, endovascular thrombectomy; mRS, modified Rankin Scale; OR, odds ratio. **P* value < 0.05.

**References**

1.	Powers WJ, Rabinstein AA, Ackerson T, et al. Guidelines for the Early Management of Patients With Acute Ischemic Stroke: 2019 Update to the 2018 Guidelines for the Early Management of Acute Ischemic Stroke: A Guideline for Healthcare Professionals From the American Heart Association/American Stroke Association. *Stroke*. 2019;50(12):e344-e418. doi:10.1161/STR.0000000000000211

2.	Sacco RL, Kasner SE, Broderick JP, et al. An updated definition of stroke for the 21st century: a statement for healthcare professionals from the American Heart Association/American Stroke Association. *Stroke*. 2013;44(7):2064-2089. doi:10.1161/STR.0b013e318296aeca

3.	Sebök M, Esposito G, Niftrik CHB van, et al. Flow augmentation STA-MCA bypass evaluation for patients with acute stroke and unilateral large vessel occlusion: a proposal for an urgent bypass flowchart. *J Neurosurg*. Published online January 7, 2022:1-9. doi:10.3171/2021.10.JNS21986

4.	Slessarev M, Han J, Mardimae A, et al. Prospective targeting and control of end-tidal CO2 and O2 concentrations. *J Physiol*. 2007;581(3):1207-1219. doi:https://doi.org/10.1113/jphysiol.2007.129395

5.	Sebök M, Niftrik CHB van, Piccirelli M, et al. BOLD cerebrovascular reactivity as a novel marker for crossed cerebellar diaschisis. *Neurology*. 2018;91(14):e1328-e1337. doi:10.1212/WNL.0000000000006287

6.	Liu CF, Hsu J, Xu X, et al. Digital 3D Brain MRI Arterial Territories Atlas. *Sci Data*. 2023;10(1):74. doi:10.1038/s41597-022-01923-0

7.	Bellomo J, Sebök M, Stumpo V, et al. Blood Oxygenation Level–Dependent Cerebrovascular Reactivity–Derived Steal Phenomenon May Indicate Tissue Reperfusion Failure After Successful Endovascular Thrombectomy. *Transl Stroke Res*. Published online October 25, 2023. doi:10.1007/s12975-023-01203-y

8.	Bellomo J, Sebök M, van Niftrik CH, et al. The volume of steal phenomenon is associated with neurological deterioration in patients with large-vessel occlusion minor stroke not eligible for thrombectomy. *Eur Stroke J*. Published online May 14, 2024:23969873241251718. doi:10.1177/23969873241251718

9.	Sebök M, van Niftrik CHB, Piccirelli M, et al. Crossed Cerebellar Diaschisis in Patients With Symptomatic Unilateral Anterior Circulation Stroke Is Associated With Hemodynamic Impairment in the Ipsilateral MCA Territory. *J Magn Reson Imaging JMRI*. 2021;53(4):1190-1197. doi:10.1002/jmri.27410

10.	Liu CF, Hsu J, Xu X, et al. Deep learning-based detection and segmentation of diffusion abnormalities in acute ischemic stroke. *Commun Med*. 2021;1:61. doi:10.1038/s43856-021-00062-8
